# Quality Traceability System of Traditional Chinese Medicine Based on Two Dimensional Barcode Using Mobile Intelligent Technology

**DOI:** 10.1371/journal.pone.0165263

**Published:** 2016-10-25

**Authors:** Yong Cai, Xiwen Li, Runmiao Wang, Qing Yang, Peng Li, Hao Hu

**Affiliations:** 1 State Key Laboratory of Quality Research in Chinese Medicine, Institute of Chinese Medical Sciences, University of Macau, Macao, China; 2 Information Technology College, Beijing Normal University Zhuhai Campus, Zhuhai, China; 3 Institute of Chinese Materia Medica, China Academy of Chinese Medical Sciences, Beijing, China; 4 State Key Laboratory of Hydraulics and Mountain River Engineering, Sichuan University, Chengdu, China; Chinese Academy of Medical Sciences and Peking Union Medical College, CHINA

## Abstract

Currently, the chemical fingerprint comparison and analysis is mainly based on professional equipment and software, it’s expensive and inconvenient. This study aims to integrate QR (Quick Response) code with quality data and mobile intelligent technology to develop a convenient query terminal for tracing quality in the whole industrial chain of TCM (traditional Chinese medicine). Three herbal medicines were randomly selected and their chemical two-dimensional barcode (2D) barcodes fingerprints were constructed. Smartphone application (APP) based on Android system was developed to read initial data of 2D chemical barcodes, and compared multiple fingerprints from different batches of same species or different species. It was demonstrated that there were no significant differences between original and scanned TCM chemical fingerprints. Meanwhile, different TCM chemical fingerprint QR codes could be rendered in the same coordinate and showed the differences very intuitively. To be able to distinguish the variations of chemical fingerprint more directly, linear interpolation angle cosine similarity algorithm (LIACSA) was proposed to get similarity ratio. This study showed that QR codes can be used as an effective information carrier to transfer quality data. Smartphone application can rapidly read quality information in QR codes and convert data into TCM chemical fingerprints.

## Introduction

The industrial chain of traditional Chinese medicine is a complicated process including multiple steps. The quality and safety issues of traditional Chinese medicine have plagued the scientists for many years. It’s necessary to ensure the quality to be safe, effective, stable and controllable in each link of the whole process, especially in production and circulation of Chinese herbal medicine which is the origin of TCM. Unfortunately almost all current traceability technologies can only provide production or sale information in circulation with no quality data. Chemical fingerprints of TCM is an important quality control models and technologies to identify the quality of traditional Chinese medicines [1–3]. The success of conversion from chemical fingerprint to two-dimensional barcode has created an opportunity to develop a traceability system delivering quality information [4]. However how to apply quality two-dimensional barcode to practical quality traceability in circulation and personal consumed terminal still remains elusive.

QR code has been used in traceability systems of fish [5] and also was applied to vegetable [6] traceability combined with mobile service. Currently combining smartphones power sensors and cloud computing technologies, scientists have tried to build a mobile health care system that could benefit the public [7–9]. The sensor capabilities were also used to allow smartphones to provide location based services and gathered data from camera or QR code by scanning [10–12]. Mobile intelligent technology and two-demensional code scanning provided probability to trace good quality or bad of TCM in circulation and personal consumer terminal. By the end of 2011, there were over 130 million people who used iPhone, iPad or iTouch in the world and the number increased 103% than that in 2010. According to a new report released by Gartner in the third quarter of 2013 [13], the number of customers using Samsung and Google's Android system exceeded that of people operating Apple and its iOS system. Hundreds of applications including developed games [14,15], booking [16], traveling [17–19] and payment [20] were installed in smartphone using Android system. Especially, the mobile health applications enabled the doctors to interact with the patients by “wang-wen-wen-qie” or monitor people to improve their life quality [21].

Currently, the chemical fingerprint comparison and analysis is mainly based on professional equipment and software, it’s expensive and inconvenient. This study aims to integrate QR code with quality data and smartphone to develop a convenient query terminal for tracing quality in the whole industrial chain of TCM. An application based on Android system was developed to recognize and analysis the chemical QR codes of Chinese medicine by scanning QR codes. We demonstrated that this APP could distinguish different batches of Chinese herbal medicine of the same species and identify the quality of different species by scanning chemical two-dimensional QR codes.

## Materials and Methods

### Materials

Three kinds of Chinese herbal medicines, YinYangHuo (Epimedium), RouCongRong (Cistanche deserticola) and MuDanPi (Cortex Moutan), were randomly selected and their chemical fingerprints were constructed using HPLC technology (Yin-YangHuo and RouCongRong refer to [22–23], resp.). The detailed method of MuDanPi detection was as follows: a Zorbax SB-C18 column (250 × 4.6mm I.D., 5 μm) with a Zorbax SB-C18 guard column (12.5 × 4.6mm I.D., 5 μm) was used. The samples were separated using a gradient mobile phase consisting of 0.5% acetic acid (A) and acetonitrile (B). The gradient condition is 0–40min, 10%–50% B; 40–60 min, 50%–100% B; 60–65min, 100%B. The separation was performed on an Agilent series 1200 liquid chromatography (Agilent Technologies, Santa Clara, CA, USA), equipped with a vacuum degasser, a quaternary pump, an autosampler, and a diode array detector (DAD). The chromatographic analysis method for chemical profiling refer to [24]. Testing data of chemical fingerprints were converted into 2D barcodes by online website (http://qrgenerator.qrcreator.net) [4].

### System environment and smartphone application development

Android is an operating system based on Linux kernel. Android smartphone was used in this study. Development environment is shown in Table 1.

**Table 1:** Development environment.

**JDK**	JDK 1.7.0_45
**Android SDK**	Android 4.3
**Barcode image processing library for Android**	Google Zxing 1.6
**Charting library for Android**	AchartEngine 1.1.0
**Android Developer Tools**	ADT Build: v22.3.0–887826
**Operation System**	Window XP(Service Package 3)
**Mobile phone (OS)**	Samsung Galaxy mini with Android with OS version 2.3

**Fig 1:**
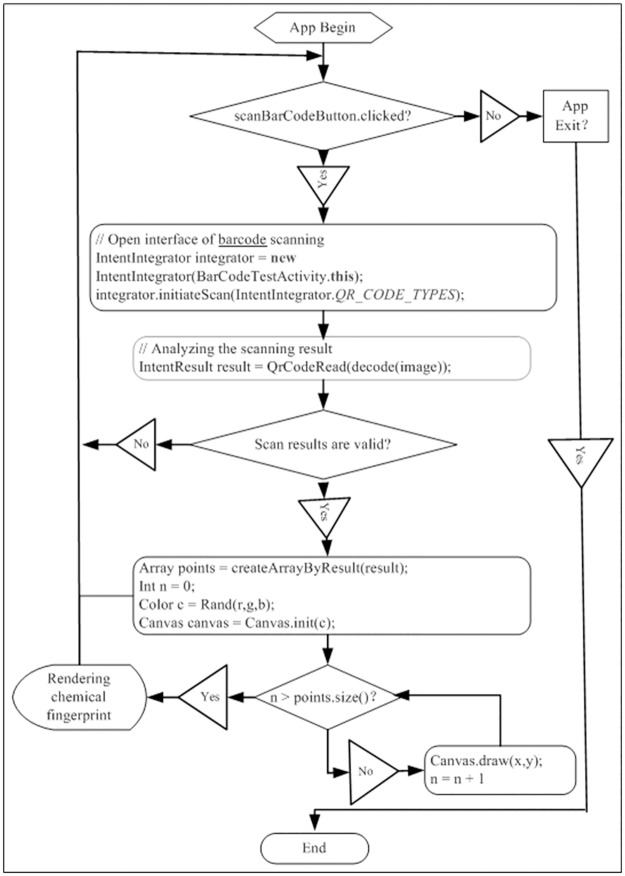
Whole process of application based on Android system.

APP were coded by Android Developer Tools. The Android Package (APK) was installed on Android smartphone. The whole processes of application based on Android system are shown in Fig 1.

### Scan of QR codes and presentation of TCM chemical fingerprints

Chart of chemical fingerprint was generated by plug-in AchartEngine, an open source graphics library (Google). Line chart was adopted to form chemical fingerprints.

**Fig 2:**
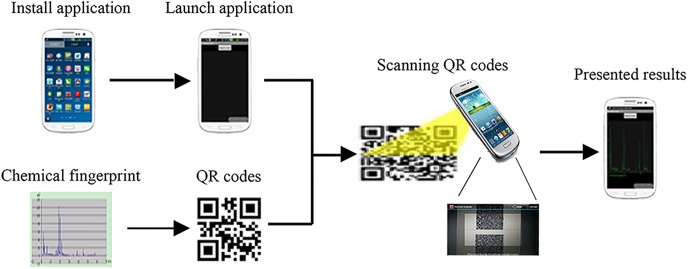
Flow chart of recognition of QR codes to restore chemical fingerprints.

Different chemical QR codes were scanned and results were shown in the same screen. Chemical fingerprints are designed with different colors. Different chemical fingerprints of Chinese herbal medicines were compared to intuitively find their difference by scanning QR codes one by one. The flow chart of recognition of QR codes to restore chemical fingerprints was shown in Fig 2.

### Chemical fingerprint similarity comparison algorithm

**Fig 3:**
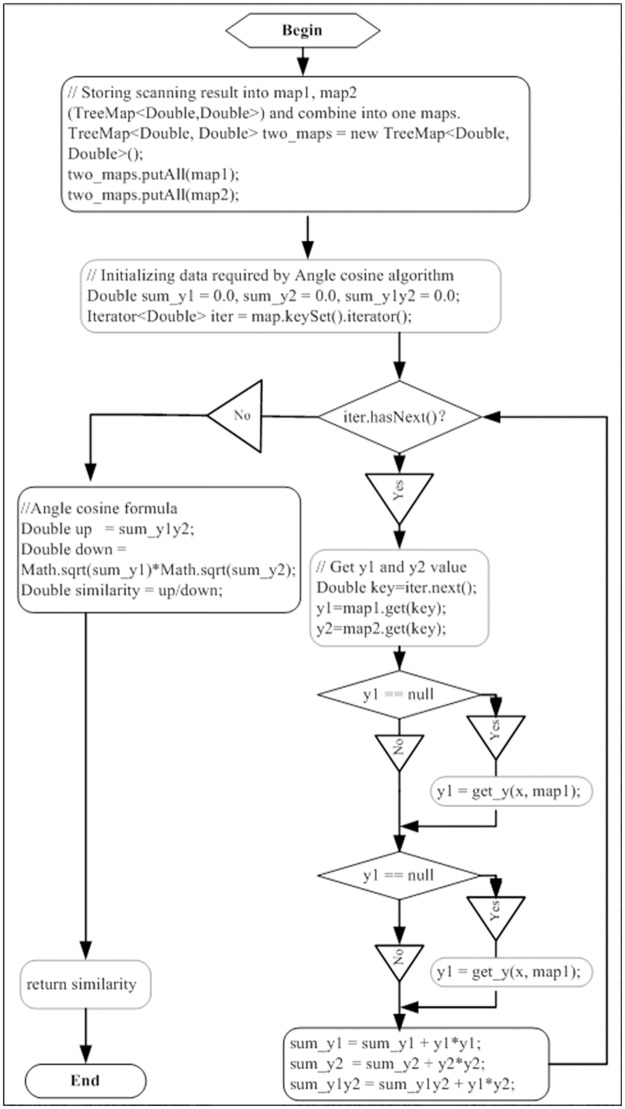
Linear interpolation angle cosine similarity algorithm.

**Fig 4:**
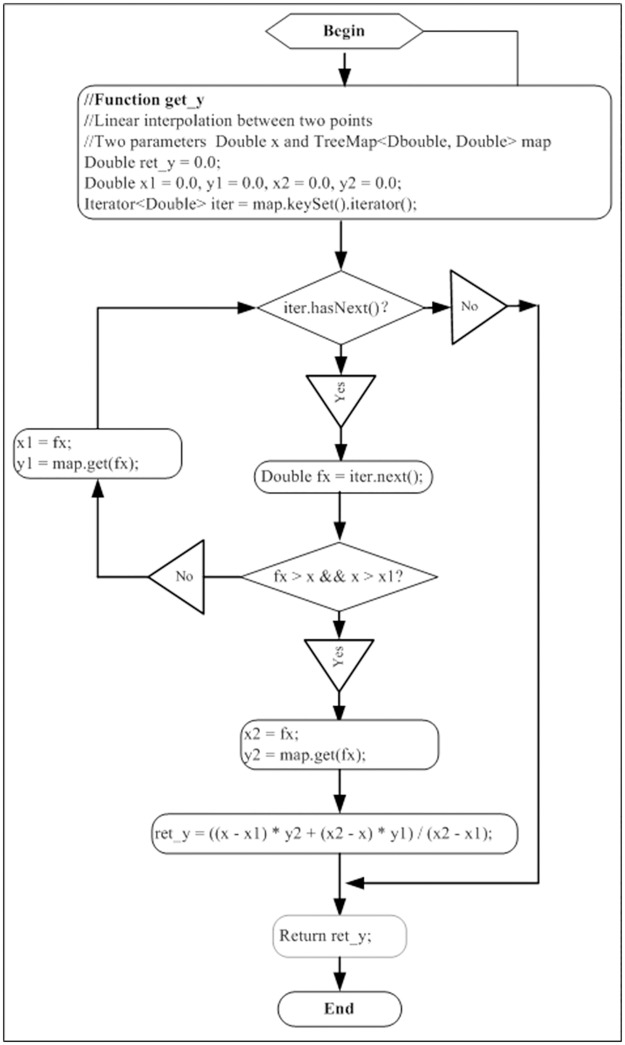
Linear interpolation function.

To be able to quantitatively distinguish the difference between chemical fingerprints, this experiment proposed LIACSA to obtain quantitative similarity, as shown in Figs 3 and 4.

## Results and Discussion

Quality traceability plays an important role to ensure TCM to be safe, effective, stable and controllable from the point of production to the point of consumption. However, most current traceability systems are paper files or electronic-based tags. They typically rely on physical barcodes attached to products ostensibly tracing the products from stages of the production process, and integrity of traceability trail is only as reliable as the reading and transcription of the data. New technologies linked to genomics and informatics that involve gene level identifiers show promise in improving both the herbal identification and cost of regulatory oversight. For example, DNA barcoding was used for traceability [25] and recently has been admitted to the Chinese Pharmacopoeia as one of the methods for species identification (Chinese Pharmacopoeia, 2015). Some scholars have conducted research on how to convert DNA sequences into the 2D barcode for quality control. Kumar et al and Liu et al carried on studies on encoding type of DNA 2D barcode respectively [26,27]. In addition, Liu et al proposed that the technology of converting fingerprint into 2D barcode could be applied to the identification. However no current traceability methods can provide quality information so that we cannot identify the attribute of TCM without the data from suppliers. Chemical fingerprinting was a commonly used quantified testing method. However, it was not amenable to information storage, recognition and retrieval. Cai et al converted chemical fingerprint of TCM into data format that could be stored as QR code through data preprocessing [4], which made the transformation of quality information possible. In this study, we made a further attempt on how to apply 2D barcode of chemical fingerprint to quality traceability in actual production and circulation.

**Fig 5:**
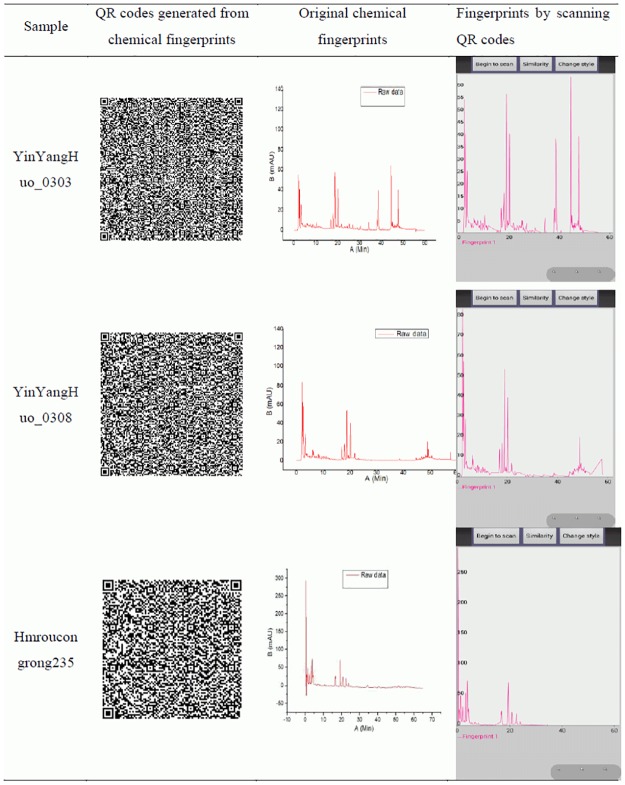
Comparison of scanning results and initial chemical fingerprint of four batches.

According to the method reported by Yong Cai et al [4], chemical fingerprints were successfully converted into 2D barcodes and QR codes were selected as coding type (Fig 5). The aim of this study focused on how to extract data from chemical 2D barcodes and generate chemical fingerprints to evaluate the quality of TCM by consumer smartphone. We firstly developed an application (Fig 1) based on Android platform which installed in smart phone. Four chemical 2D barcodes were scanned and quality data were extracted. Using this APP, four different chemical fingerprints were re-generated (Fig 5).

**Fig 6:**
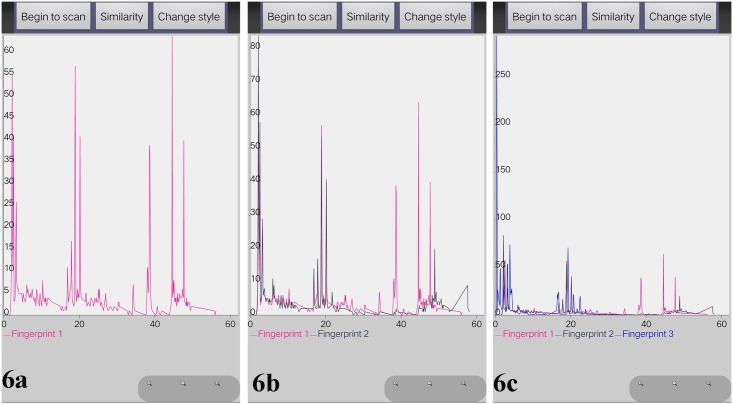
Multi-curve rendering of different TCM chemical fingerprints. 6a,YinYangHuo_0303 (In pink color); 6b, YinYangHuo_0303 (In pink color) / YinYangHuo_0308 (In navy color); 6c, YinYangHuo_0303 (In pink color) / YinYangHuo_0308 (In navy color) / Hmroucongrong235(In mid-blue color).

**Fig 7:**
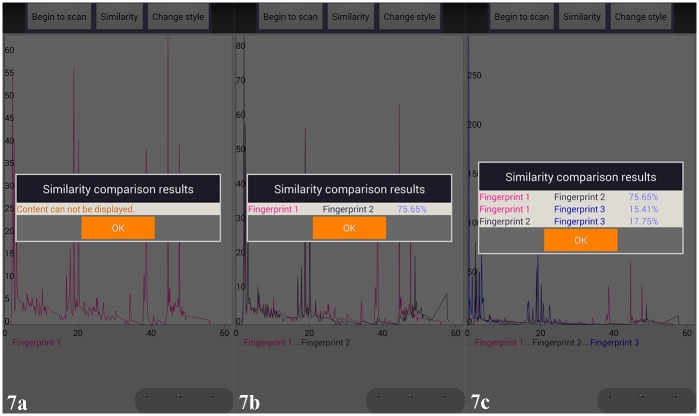
Similarity comparison results of TCM chemical fingerprints. 7a, YinYangHuo_0303; 7b, YinYangHuo_0303 (In pink color) / YinYangHuo_0308 (In navy color); 7c, YinYangHuo_0303 (In pink color) / YinYangHuo_0308 (In navy color) / Hmroucongrong235(In mid-blue color).

**Fig 8:**
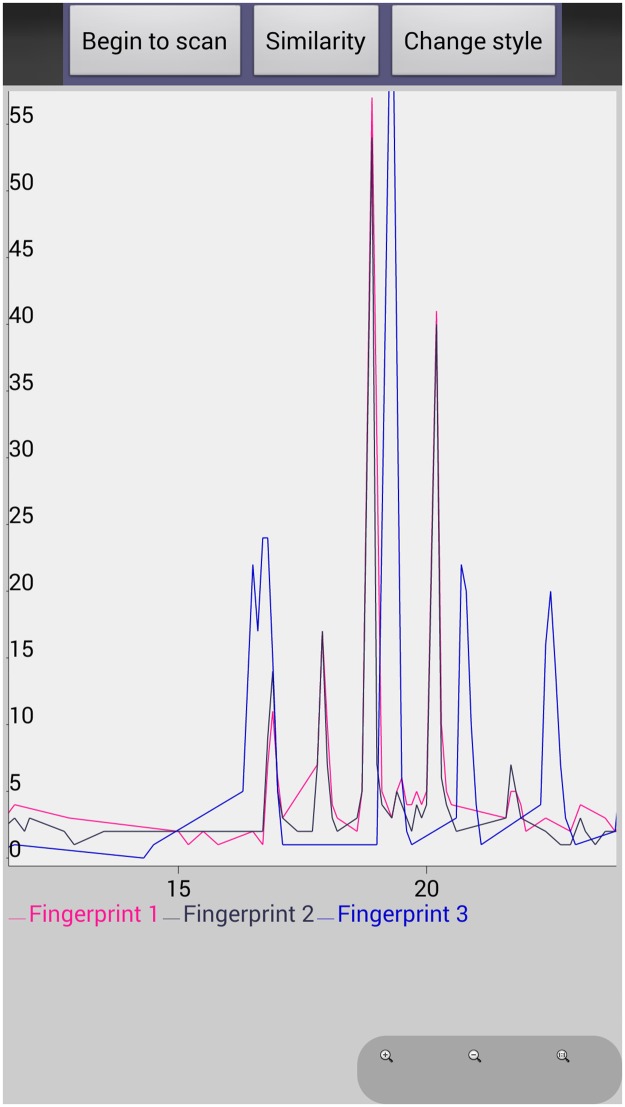
Comparing local details between chemical fingerprints by ‘zoom’ function.

**Fig 9:**
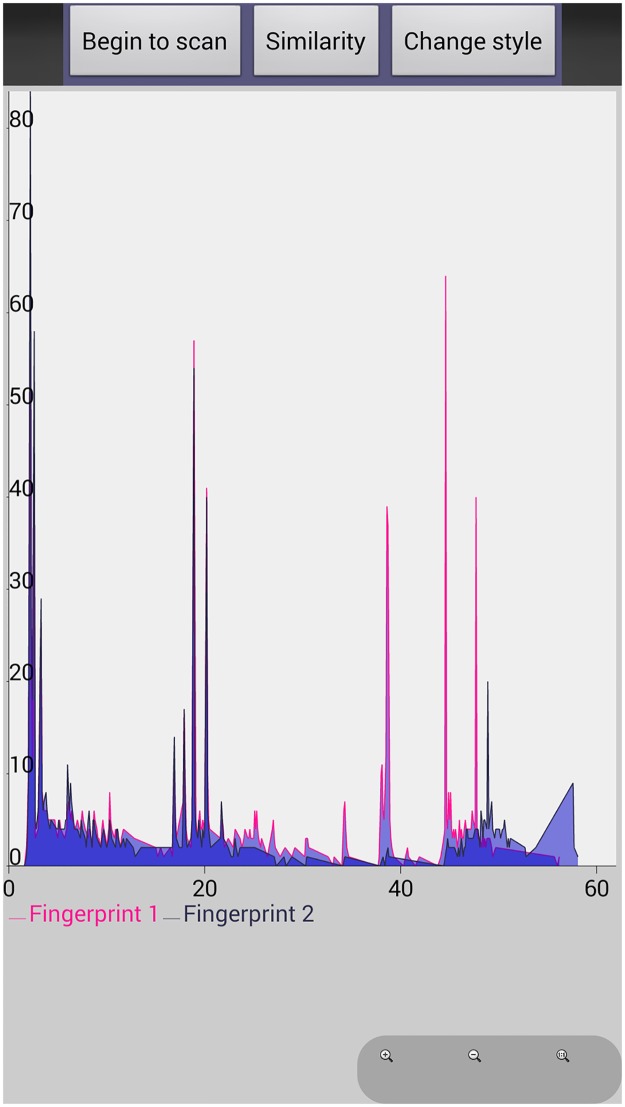
Comparing peak area between chemical fingerprints by ‘change style’ function.

After comparison between the original and newly generated chemical fingerprints, we found that they had the same characteristic peaks which represented different chemical compounds. In addition. We have developed additional functionality on this APP including comparing different chemical fingerprints on the same screen and same coordinate system by scanning different 2D barcodes (Fig 6), and proposed LIACSA algorithm to obtain similarity ratio directly between chemical fingerprints (Fig 7b and 7c); local details comparison by ‘zoom’ function (Fig 8); peak area comparison between chemical fingerprints by ‘Change style’ function (Fig 9). It could compare different chemical 2D barcodes from different batches of the same Chinese herbal medicine (Figs 6b and 7b) or different Chinese herbal medicines (Figs 6c and 7c).

With these functions, researchers and consumers can scan multiple 2D barcodes continuously and get their chemical fingerprints to evaluate their quality intuitively. From Figs 6 and 7, we found YinYangHuo_0303 and YinYangHuo_0308 might be the same Chinese material medica but from different batches (More than 75% similarity), YinYangHuo_0303 and Hmroucongrong235 might be from different herbal medicines (Less than 16% similarity). This APP showed their differences visually and directly, which was helpful for producers and consumers to determine the quality in process and market. Therefore the quality of each batch of herbal medicine could be tracked using this APP installed in smart phones.

There are many similarity algorithms for chemical fingerprint, such as angle cosine, Pearson correlation coefficient and Euclidean distance and so on [28]. This experiment proposed LIACSA (Fig 4), which added linear interpolation based on cosine similarity algorithm. This algorithm is applicable to the case when one of Y-axis value missed between two chemical fingerprints.

This study demonstrated that smartphone technology can restore chemical fingerprint of TCM by scanning chemical 2D barcodes, and maintain consistency with initial chemical fingerprint generated from HPLC software. This research will provide consumers and managers an easy and rapid way to trace the quality in circulation and management of traditional Chinese medicine.

## Conclusions

Smart phone application can be developed to provide a convenient way for producers, mangers and consumers to trace the quality of traditional Chinese medicine. It can convert quality data of TCM into corresponding chemical fingerprints by scanning QR codes and shows the difference between herbal medicines intuitively. This study provide an effective tool for regulating each link in production chain of traditional Chinese medicine by establishing quality traceability system in the future.
